# 2′-SCF3 Uridine–A Powerful Label for Probing Structure and Function of RNA by 19F NMR Spectroscopy**

**DOI:** 10.1002/anie.201207128

**Published:** 2012-11-19

**Authors:** Katja Fauster, Christoph Kreutz, Ronald Micura

**Affiliations:** Institute of Organic Chemistry (IOC) and Center for Molecular Biosciences (CMBI), University of Innsbruck, Center for Chemistry and Biomedicine (CCB)020 Innsbruck (Austria)

**Keywords:** nucleosides, riboswitch, RNA, solid-phase synthesis, trifluoromethylation

Magnetic resonance methods that employ the ^19^F nucleus for cellular imaging or molecular structure and dynamics investigations become increasingly important for both in vitro and in vivo systems.^1^ Fluorine is hardly encountered in biomolecules and therefore provides excellent bioorthogonality. However, what is an advantage on the one hand, can become an obstacle on the other hand because proper methods for labeling are required. Although an elegant alternative that relies on “spy molecules”, which contain the fluoride sensor, has been reported recently,^2^ direct labeling of either the target or the interaction partner remains inevitable for the majority of successful applications.

Our group has a strong focus on ^19^F labeling of RNA in order to utilize the corresponding derivatives for structural and functional analysis.^3^ For instance, we disclosed a gene-regulation-determining, bistable sequence element in the preQ_1_ class I riboswitch based on strategically positioned 5-F uridine labels in the corresponding mRNA domain.^4^ In another example, we introduced ribose 2′-F atoms at specific nucleoside positions, allowing local monitoring of binding events and thus visualizing dynamic RNA–ligand interactions.^5^ Although being powerful, in all these cases, the reporter unit relied on a single fluorine atom, and thus limitations with respect to sensitivity could potentially be encountered.

Herein, we present a novel high-performance fluorine sensor for RNA, the ribose 2′-deoxy-2′-trifluoromethylthio unit (2′-SCF_3_; Figure 1). The advantage of this label lies in the fact that three magnetically equivalent fluorine atoms allow ^19^F NMR experiments to be performed at micromolar concentrations. This labeling method thus constitutes a significant improvement compared to the above-mentioned single-atom labels, which require RNA concentrations in the low millimolar range; less material is needed and potential aggregation problems are minimized. Moreover, the 2′-SCF_3_ group represents an isolated spin system, therefore proton decoupling (as, for example, required for 2′-F labels) is not necessary, thus making the label metrologically very straightforward. Similar to methyl groups, trifluoromethyl groups allow the prolongation of coherence lifetime based on transverse relaxation optimized spectroscopy (TROSY), accounting for an additional advantage in measurements of large RNA molecules or RNA–protein systems.

**Figure 1:**
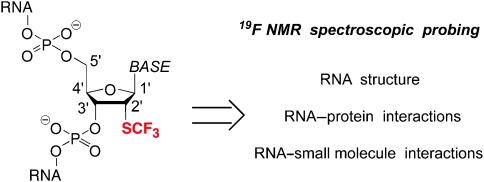
New concept for fluorine labeling of RNA with respect to ^19^F NMR spectroscopic applications.

Originally, we considered to develop a new ^19^F label for RNA applications through trifluoromethylation.^6^ The selection of an appropriate nucleoside position is critical because several sites are to be excluded. For example, 5-trifluoromethyl uridine is chemically unstable during oligonucleotide deprotection as it can transform into a 5-cyano group.^7^ The 5 position of uridine can be functionalized by the sterically demanding 4,4,4-trifluoro-3,3-bis(trifluoromethyl)butyne residue, and this pyrimidine label with nine equivalent fluorine atoms was successful in ^19^F NMR spectroscopic analysis of DNA hybridization.^8^ However, we did not pursue such a concept in favor of a uniform labeling pattern at sites that are equivalent in all four standard nucleosides, preferably at the 2′ position. In this sense, the logical follow-up consideration was trifluoromethylation of the 2′ hydroxy group of ribose to achieve 2′-OCF_3_ labels. To our knowledge, a single study on 2′-OCF_3_-modified oligonucleotides has been reported to date, and this refers to 2′-OCF_3_ adenosine.^9^ Introduction of the modification was achieved via 2′-*O*-[(methylthio)thiocarbonyl]adenosine by treatment with pyridinium poly(hydrogen fluoride) (HF/pyridine) in the presence of 1,3-dibromo-5,5-dimethylhydantoin (DBH), however, yields were extremely low (22 %). In our own attempts, we were unable to increase the reported yields. Efforts to apply a new class of electrophilic trifluoromethylation reagents based on hypervalent iodine(III) derivatives (Togni reagents)^10^ for the zinc-mediated trifluoromethylation of the 2′-OH group of a 5′,3′-O-protected guanosine substrate failed. Furthermore, by using the first mentioned xanthate method,^11^ we were able to generate minor amounts of 2′-OCF_3_ uridine derivative. However, this pyrimidine nucleoside turned out to be unstable, its decomposition resulting in formation of 2,2′-anhydrouridine.

These observations prompted us to develop a novel concept for RNA labeling, namely with 2′-SCF_3_ nucleosides, which appears rather unorthodox at first sight. Although such a label would most likely thermodynamically destabilize an RNA double helix (assuming that its behavior would be analogous to 2′-SCH_3_ residues),^12^ the many promising ^19^F NMR applications for probing structure and folding of RNA, binding of small molecules and RNA, or protein–RNA interactions, for which this label can be easily positioned in single-stranded regions, prompted us to pursue this goal.

We started our endeavors with 2′-deoxy-2′-mercaptouridine **1** (Scheme 1) which is readily accessible in large amounts from 2,2′-anhydrouridine and thioacetic acid, according to an early report.^13^ Fortunately, the key step of our synthetic plan, the regioselective trifluoromethylation of the thiol group, was achieved in 80 % yield using 3,3-dimethyl-1-(trifluoromethyl)-1,2-benziodoxole (Togni’s reagent).^14^ The trifluoromethylated thiouridine **2** was completely stable and no back reaction to 2,2′-anhydrouridine was observed (contrarily to the 2′-OCF_3_ counterpart). Subsequently, the 5′-OH group was protected as dimethoxytrityl (DMT) ether to give compound **3**, and conversion into the corresponding phosphoramidite **4** was achieved in good yield by reaction with 2-cyanoethyl *N*,*N*-diisopropylchlorophosphoramidite. Starting from compound **1**, our route provides **4** in 60 % overall yield in three steps with three chromatographic purifications; in total, 1.9 g of **4** was obtained in the course of this study.

**Scheme 1:**
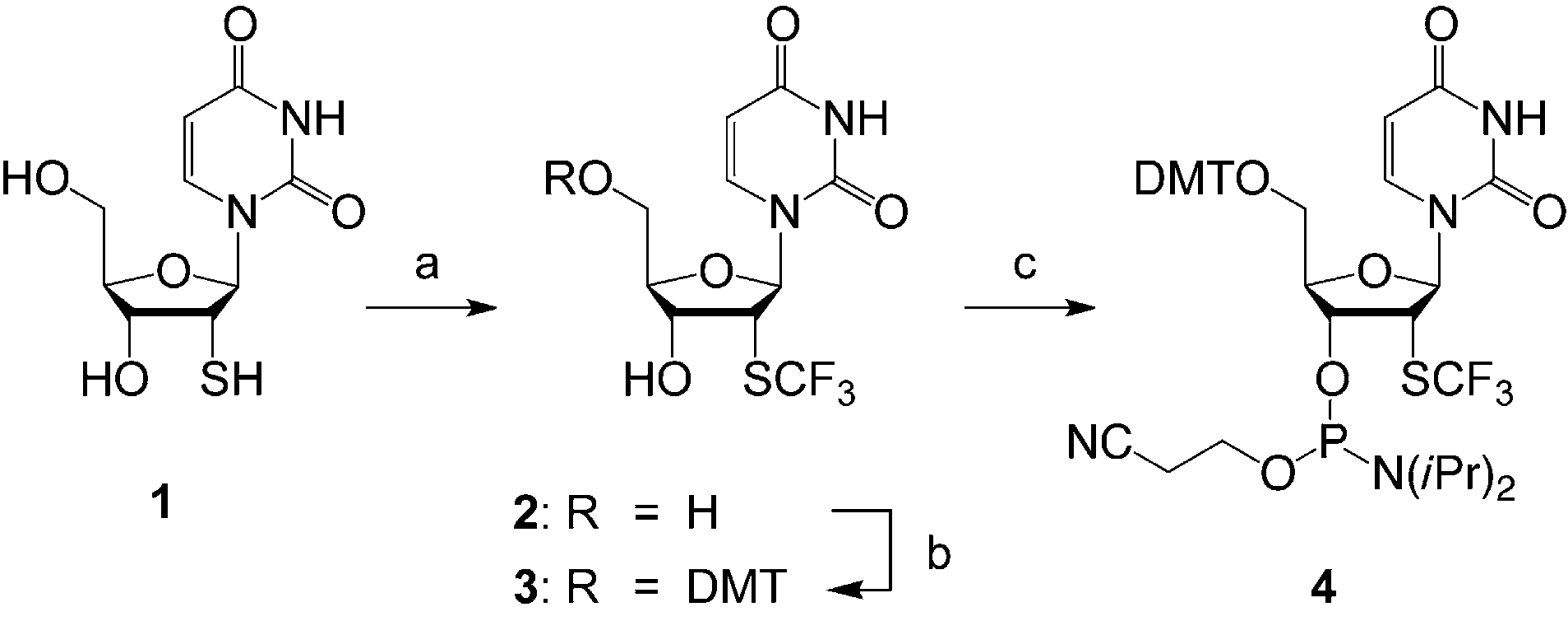
Synthesis of the 2′-deoxy-2′-trifluoromethylthio (2′-SCF_3_) uridine building block 4 for RNA solid-phase synthesis. Reagents and conditions: a) 3,3-dimethyl-1-(trifluoromethyl)-1,2-benziodoxole (1.2 equiv), CH_3_OH, −78 °C→RT, 16 h, 80 %; b) DMT-Cl (1.1 equiv), DMAP (0.1 equiv), pyridine, RT, 16 h, 81 %; c) (2-cyanoethyl)-*N*,*N*-diisopropyl chlorophosphoramidite (1.5 equiv), *N*-ethyldiisopropylamine (10 equiv), CH_2_Cl_2_, RT, 2.5 h, 93 %.

Next, the preparation of RNA with the novel 2′-SCF_3_ uridine building block was tested, using the solid-phase synthesis methodology for 2′-*O*-TOM-protected RNA.^15^ Coupling yields were higher than 98 % according to the trityl assay. Cleavage from the solid support and deprotection of the modified RNA molecules were performed in the presence of CH_3_NH_2_ in ethanol/H_2_O, followed by treatment with tetrabutylammonium fluoride (TBAF) in tetrahydrofuran (THF). Salts were removed by size-exclusion chromatography on a Sephadex G25 column, and RNA sequences were purified by anion-exchange chromatography under strong denaturating conditions (6 m urea, 80 °C; Figure 2). The molecular weights of the purified RNA molecules were confirmed by liquid-chromatography (LC) electrospray-ionization (ESI) mass spectrometry (MS). Synthesized RNA sequences containing 2′-SCF_3_ uridine labels are listed in Table 1 in the Supporting Information. Noteworthy, the 2′-SCF_3_ label was completely stable under repetitive oxidative conditions (20 mm aqueous iodine solution) required during RNA solid-phase synthesis for transformation of P^III^ to P^V^. Therefore, no special adaptions of the standard synthesis cycle (as, for example, required for 2′-SeCH_3_-modified RNA)^16^ were necessary to provide high-quality crude products (Figure 2).

**Figure 2:**
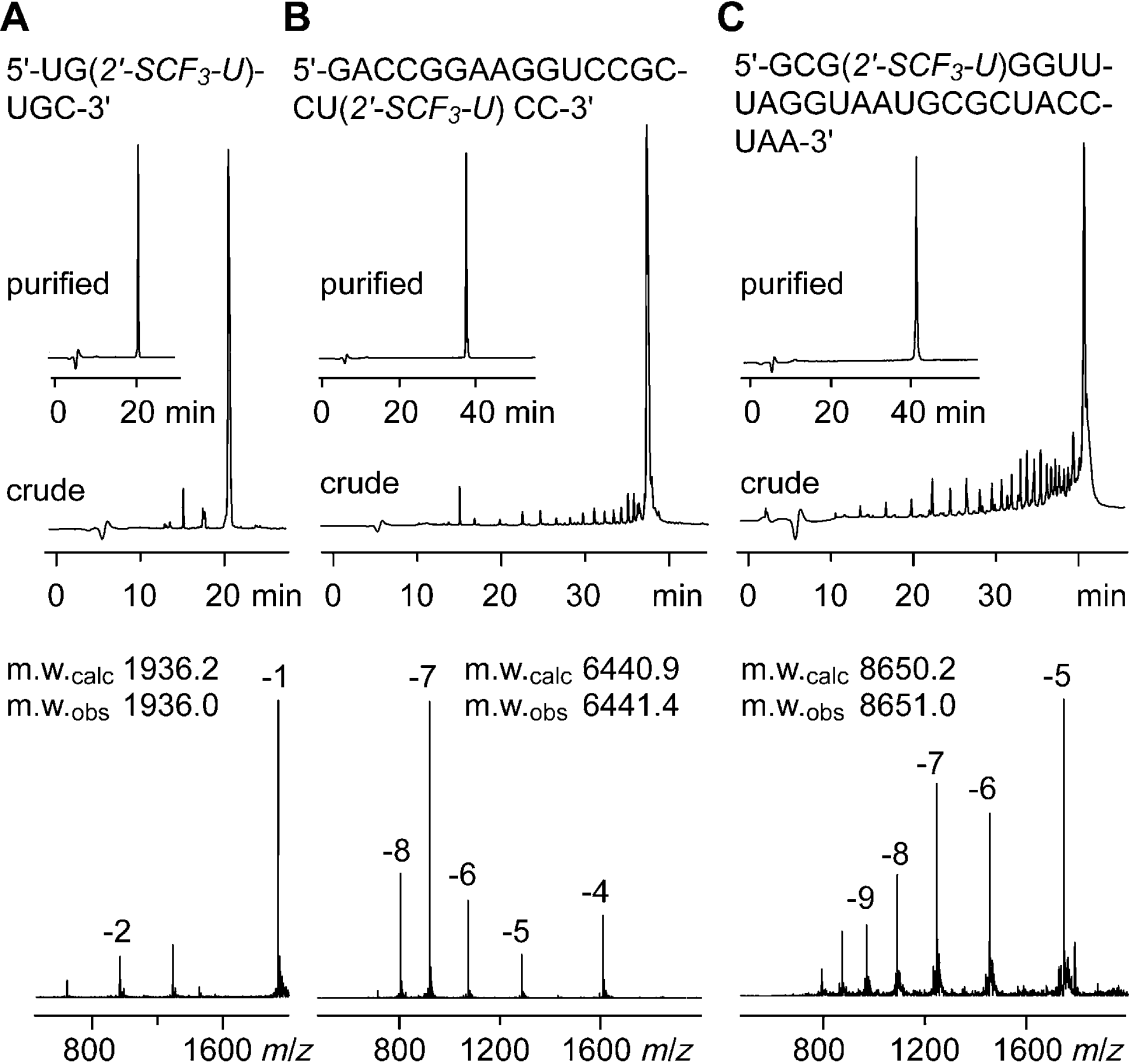
Characterization of 2′-SCF_3_ modified RNA. Anion-exchange HPLC traces (top) of 6 nt RNA (A), 20 nt RNA (B), and 27 nt RNA (C), and respective LC-ESI mass spectra (bottom). HPLC conditions: Dionex DNAPac column (4×250 mm), 80 °C, 1 mL min^−1^, 0→60 % buffer B in 45 min; buffer A: Tris-HCl (25 mm), urea (6 m), pH 8.0; buffer B: Tris-HCl (25 mm), urea (6 m), NaClO_4_ (0.5 m), pH 8.0. For LC-ESI MS conditions, see the Supporting Information.

The efficient synthetic access to 2′-SCF_3_-modified RNA encouraged us to evaluate the new label in ^19^F NMR applications. In the following, we present three examples: 1) probing of the secondary structure of bistable RNA sequences; 2) verification of RNA–protein interactions; and 3) attesting rationally designed riboswitch modules.

Figure 3 depicts a 32 nt long RNA sequence (**5**) that exists in slow conformational exchange of two distinct secondary structures (**5′** and **5′′**), as confirmed by comparative imino proton NMR spectroscopy^18^ using the truncated reference hairpin **5 a**. When we labeled this RNA with 2′-SCF_3_ at uridine-26 (**6**), the label lies within a 4 nt loop of fold **6′** while it is located in a 7 nt internal bulge of fold **6′′**. The assignment of secondary structures **6′** and **6′′** by ^19^F NMR spectroscopy is depicted in Figure 3 B and provides a ratio of about 55:45 in favor of **6′**. The imino proton NMR spectra of modified (**6**) and unmodified (**5**) sequences are nearly identical, thus demonstrating that the equilibrium position has not been influenced by the label. This tendency was confirmed for a second bistable RNA (see the Supporting Information, Figure 1) and hence underscores the applicability of the label for secondary-structure probing. Even more satisfying was the observation that in *E. coli* lysate at a very low RNA concentration of 10 μm, the two folds were readily detectable (Figure 3 D), showing the potential of the 2′-SCF_3_ label for in vivo studies. In this context, we should mention that the modification is likely to improve resistance against phosphodiesterases, as has been shown for the 2′-OCF_3_ counterparts.^9^

**Figure 3:**
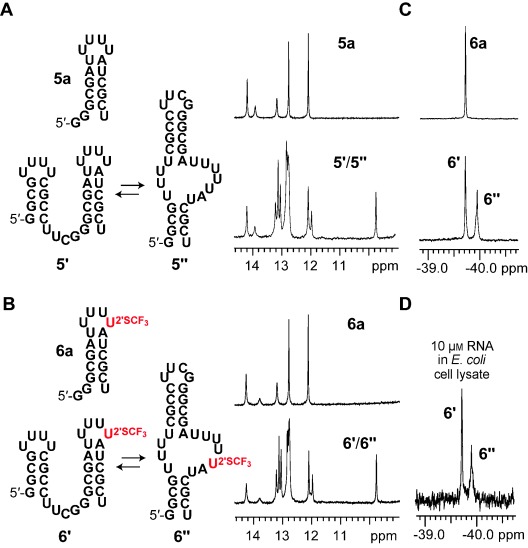
Structure probing of a bistable RNA. A) Unmodified RNA;^17^ secondary structure model of full-length (5) and reference (5 a) RNA (left); imino proton NMR spectra (right). B) Same as (A), but for 2′-SCF_3_ labeled analogues. C) Assignment of folds 6′ and 6′′ of RNA 6 by ^19^F NMR spectroscopy. D) Same as C, but in *E. coli* cell lysate. Conditions for A–C: [RNA]=0.3 mm, [Na_2_HAsO_4_]=25 mm, pH 7.0, H_2_O/D_2_O=9:1, 298 K; conditions for D: [RNA]=10 μm, *E. coli* lysate/D_2_O=9:1, 298 K (for lysate preparation, see the Supporting Information).

As a second example, we demonstrate the utility of the 2′-SCF_3_ label for the verification of RNA–protein interactions. We synthesized the stem–loop RNA molecules **7** and **8**, which comprise the recognition sequence for the small nuclear ribonucleoprotein U1A (Figure 4).^19^ The RNA-binding domain (U1A-RBD) of this protein binds to its cognate RNA with an apparent *K*_d_ of about 2×10^−11^ m. We positioned the 2′-SCF_3_ moieties within the 10 nt loop, close to the conserved sequence of AUUGCAC (Figure 4). Hairpin **7** showed a major ^19^F NMR resonance at −39.90 ppm and a minor one up-field in the signal flank, reflecting an additional conformational population in slow exchange, most likely because of a different microenvironment in the loop (Figure 4 A). When one equivalent of U1A-RBD was added, a new uniform signal at −39.70 ppm was detected, representative for the high-affinity, conformationally well-defined RNA–protein complex. Likewise, when the label was shifted one nucleotide downstream in stem–loop **8**, the same behavior was observed (Figure 4 B), demonstrating that the responsiveness of the label is not restricted to a single site, and that it can even be positioned more distant from the interaction site.

**Figure 4:**
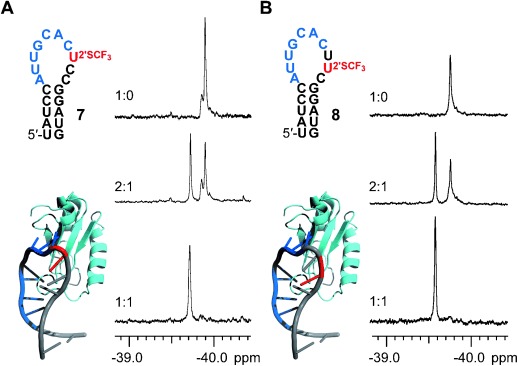
Characterization of an RNA–protein interaction. A) left: RNA stem–loop 7 with 2′-SCF_3_ label (red) and the recognition sequence (blue) for the U1 A protein (cyan), Pymol model generated from 3CUL (Protein Data Bank); right: ^19^F NMR spectra of RNA and U1 A RBD^19^ mixed at different ratios as indicated. B) Same as (A), but with label (red) at different position. Conditions: [RNA]=0.3 mm, [protein]=0.3 mm, [Na_2_HPO_4_]=10 mm, pH 6.0, H_2_O/D_2_O=9:1, 298 K.

For a third example, we designed a novel riboswitch module that consists of only 27 nucleotides and verified its function using the 2′-SCF_3_ labeling concept (Figure 5). In the emerging field of synthetic biology, such modules are of growing interest to engineer gene-regulation systems,^20^ but currently their number is rather limited and refer to only few small-molecule ligands, such as theophilline, tetracyclin, or neomycin.^21^ Here, we employed a known tobramycin-sensitive aptamer recognition sequence^22^ for the design of a novel switchable RNA module. In the free form, this functional RNA **9** exists in an extended stem–loop conformation (**9′**; Figure 5). Once tobramycin (tob) is added, it captures the minor conformation of the RNA (**9′′**) that comprises a characteristic 14 nt recognition loop to form a high-affinity complex in the nanomolar range. This implies a ligand-induced rearrangement of the secondary structure, and thus provides the typical characteristics of a riboswitch.^23^ With the focus on 2′-SCF_3_ labeling and ^19^F NMR spectroscopy, we showed that the new label allows the straightforward assignment and quantification of the different RNA conformations (**9′**, **9′′**, **9′′**-tob) involved for this riboswitch module (Figure 5 D).

**Figure 5:**
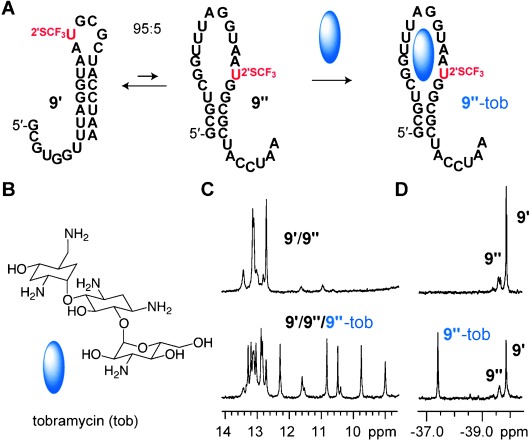
Characterization of a designed riboswitch module. A) Secondary-structure model of the proposed switchable RNA sequence 9. B) Chemical structure of tobramycin. C) Imino proton NMR spectra of RNA 9 (top) and after addition of 0.5 equivalents of tobramycin (bottom). D) Same as (C), but ^19^F NMR spectra for fold assignment. Conditions: [RNA]=0.3 mm, [Na_2_HAsO_4_]=10 mm, pH 7.0, H_2_O/D_2_O=9:1, 298 K; [tobramycin]=0.15 mm.

One issue that remains to be addressed in more detail is positioning of the label within single-stranded RNA regions. This is advisable because the attachment of 2′-SCF_3_ groups thermodynamically destabilizes RNA double helices, comparable to their 2′-SCH_3_ counterparts.12b UV melting profile analysis of two exemplary hairpins, 5′-GAAGGGCAACCUUCG and the corresponding modified RNA 5′-GAAGGGCAACC(2′-SCF_3_-U)UCG, showed a ΔΔ*G*°_298K_ of 1.9 kcal mol^−1^ (see the Supporting Information, Figure 2 and Table 2). The reason for the destabilization is most likely the preference for the C2′-endo conformation of the modified nucleoside. This theory was supported experimentally and by MD simulations for 2′-SCH_3_ moieties.12a,b To provide evidence for a comparable behavior of 2′-SCF_3_ functionalities, we synthesized a short RNA strand, 5′-UGU(2′-SCF_3_-U)GC, and determined ^3^*J* (H1′–H2′) coupling constants by 2D ^1^H,^1^H-DQF COSY NMR experiments (see the Supporting Information, Figure 3). For the 2′-SCF_3_ uridine, a value of 9.9 Hz was determined, accounting for a population of 98 % of C2′-endo ribose conformation, which is indeed a strong indication that this modification would cause interference if forced into a C3′-endo conformation, as it demands an A-form RNA double helix.^11^, ^24^

Taken together, the ^19^F NMR applications for 2′-SCF_3_-modified RNA molecules introduced here make this labeling concept a compelling new tool for probing of RNA structure and function, in particular when protein or small-molecule interaction partners are involved. Moreover, we stress that the chemical synthesis of this label is extremely robust and can be performed on large scale. Incorporation of the label into RNA is compatible with standard solid-phase synthesis and deprotection protocols, and thus highly convenient. Additionally, the potential expansion of the labeling concept to all four standard nucleosides holds promise for flexible and widespread applications in order to explore structure and dynamics of biologically relevant RNA sequences through in vitro and in vivo ^19^F NMR spectroscopic methods.

## Notes

Detailed facts of importance to specialist readers are published as ”Supporting Information”. Such documents are peer-reviewed, but not copy-edited or typeset. They are made available as submitted by the authors.
