# Supplementary material for: 2′-SCF3 Uridine–A Powerful Label for Probing Structure and Function of RNA by 19F NMR Spectroscopy**
Source: Angew Chem Int Ed Engl. 2012 Nov 19;51(52):13080–4. doi: 10.1002/anie.201207128 (PMC3555429; doi:10.1002/anie.201207128)
Supplement: Supplementary file 1 [file anie0051-13080-SD1.pdf]

Angewandte Chemie
Eine Zeitschrift der Gesellschaft Deutscher Chemiker

Supporting Information

© Wiley-VCH 2012

69451 Weinheim, Germany

2′-SCF_3 Uridine—A Powerful Label for Probing Structure and Function of RNA by ^{19}F NMR Spectroscopy**

*Katja Fauster, Christoph Kreutz, and Ronald Micura**

anie_201207128_sm_miscellaneous_information.pdf

Supporting Methods **Page**

1. Synthesis of 2'-SCF_3 uridine phosphoramidite building block **4** 2

1.1. Synthesis of 2'-Deoxy-2'-SCF_3-uridine (**2**) 2

NMR spectra of 2'-Deoxy-2'-SCF_3-uridine (**2**) 3

1.2. Synthesis of 5'-*O*-(4,4'-dimethoxytrityl)-2'-deoxy-2'-SCF_3-uridine (**3**) 4

NMR spectra of 5'-*O*-(4,4'-dimethoxytrityl)-2'-deoxy-2'-SCF_3-uridine (**3**) 5

1.3. Synthesis of 5'-*O*-((4,4'-Dimethoxytrityl)-2'-deoxy-2'-SCF_3-uridine 3'-*O*-(2-cyanoethyl *N,N*-diisopropylphosphoramidite) (**4**) 6

NMR spectra of 5'-*O*-((4,4'-Dimethoxytrityl)-2'-deoxy-2'-SCF_3-uridine 3'-*O*-(2-cyanoethyl *N,N*-diisopropylphosphoramidite) (**4**) 7

2. RNA synthesis, purification, and MS-, NMR-, and UV-spectroscopic measurements 8

Supporting Tables

Supporting Table 1 10

Supporting Table 2 12

Supporting Figures

Supporting Figure 1 11

Supporting Figure 2 13

Supporting Figure 3 14

References 15

1. Synthesis of 2'-SCF_3 uridine phosphoramidite building block 4

General. ^{1}H, ^{19}F, ^{13}C and ^{31}P NMR spectra were recorded on a Bruker DRX 300 MHz or Avance II+ 600 MHz instrument. The chemical shifts are referenced to the residual proton signal of the deuterated solvents: $CDCl_3$ (7.26 ppm), d_6-DMSO (2.49 ppm) for ^{1}H-NMR spectra; $CDCl_3$ (77.0 ppm) or d_6-DMSO (39.5 ppm) for ^{13}C-NMR spectra. ^{31}P-shifts are relative to external 85% phosphoric acid, ^{19}F-shifts are relative to external CCl_3F. ^{1}H- and ^{13}C-assignments were based on COSY and HSQC experiments. General mass spectrometric (MS) experiments were performed on a Finnigan LCQ Advantage MAX ion trap instrument in either negative and positive-ion mode. High-resolution mass spectra were recorded on a Bruker 7 Tesla wide bore Fourier transform - ion cyclotron resonance (FT-ICR) mass spectrometer equipped with an electrospray ionization (ESI) source. Analytical thin-layer chromatography (TLC) was carried out on Marchery-Nagel Polygram plates with fluorescent indicator. Flash column chromatography was carried out on silica gel 60 (70-230 mesh). All reactions were carried out under argon atmosphere. Chemical reagents and solvents were purchased from commercial suppliers and used without further purification. Organic solvents for reactions were dried overnight over freshly activated molecular sieves (4 Å).

1.1. Synthesis of 2'-Deoxy-2'-trifluoromethylthiouridine (2)

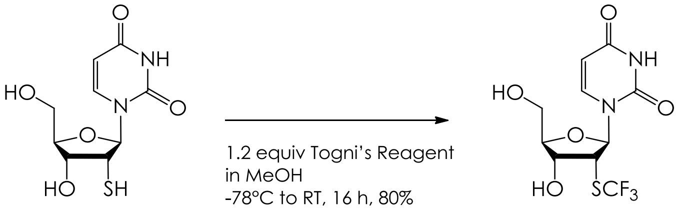

1 $C_9H_{12}N_2O_5S$ [260.27]

2 $C_{10}H_{11}F_3N_2O_5S$ [328.26]

2'-Deoxy-2'-trifluoromethylthiouridine (**2**). Compound **1**[1] (572 mg, 2.19 mmol) was suspended in methanol (12 ml) and stirred until a clear solution was obtained. The solution was cooled down to -78°C and treated with 3,3-dimethyl-1-(trifluoromethyl)-1,2-benziodoxole (870 mg, 2.64 mmol). The resulting suspension was stirred for ten minutes at -78°C under argon atmosphere, followed by stirring overnight at room temperature. Subsequently, the solvents were removed by evaporation and the crude product was purified by column chromatography on silica gel (CH_2Cl_2/CH_3OH, 98/2 – 92/8 v/v). Yield: 580 mg of **2** as white foam (80%). TLC (CH_2Cl_2/CH_3OH, 9/1): R_f = 0.50. <u>^{1}H-NMR</u> (300 MHz, d_6-DMSO): δ 3.60 (t, J = 4.1 Hz, 2H, 2 x C(5')-H); 3.97-4.02 (m, 2H, C(3')-H and C(4')-H); 4.33 (t, J = 5.2 Hz, 1H, C(2')-H); 5.26 (t, J = 5.0 Hz, 1H, C(5')-OH); 5.76 (dd, J_1 = 1.7 Hz, J_2 = 8.2 Hz, 1H, C(5)-H); 6.24 (d, J = 9.2 Hz, 1H, C(3')-OH); 6.41 (d, J = 5.6 Hz, 1H, C(1')-H); 7.88 (d, J = 8.1 Hz, 1H, C(6)-H); 11.43 (s, 1H, N(3)-H) ppm. <u>^{13}C-NMR</u> (75 MHz, d_6-DMSO): δ 50.58 (C(2')); 61.91 (C(5')); 73.23 (C(3')); 87.28 (C(4')); 87.91 (C(1')); 103.54 (C(5)); 125.32, 129.38, 133.44, 137.49 (CF_3); 140.59 (C(6)); 151.34 (C(2)); 163.35 (C(4)) ppm. <u>^{19}F-NMR</u> (564 MHz, d_6-DMSO): δ -38.94 (SCF_3) ppm. <u>FT ICR ESI-MS</u> (m/z): $[M+Na]^+$ calcd 351.023 30; found 351.023 33.

^{1}H-NMR (300 MHz, DMSO) of compound **2**:

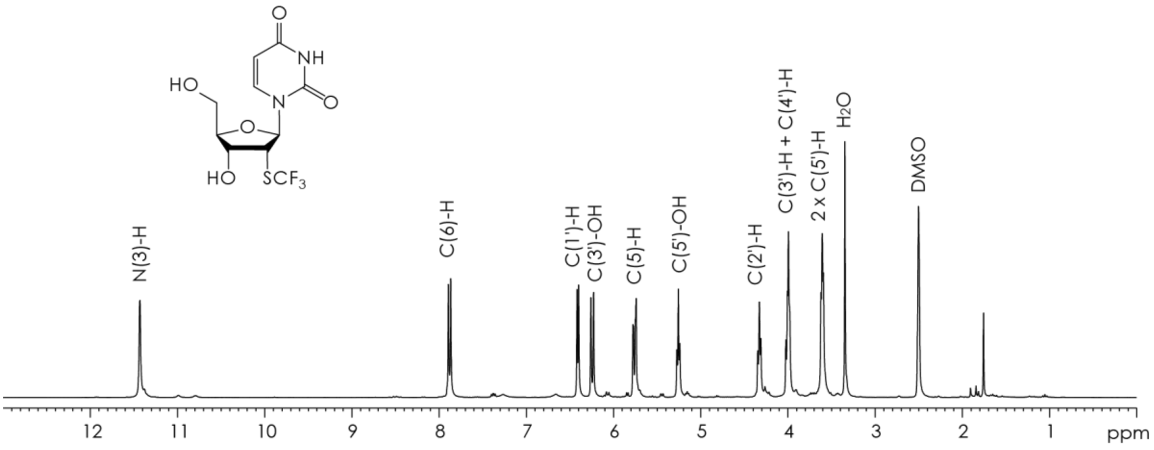

^{13}C-NMR (75 MHz, DMSO) of compound **2**:

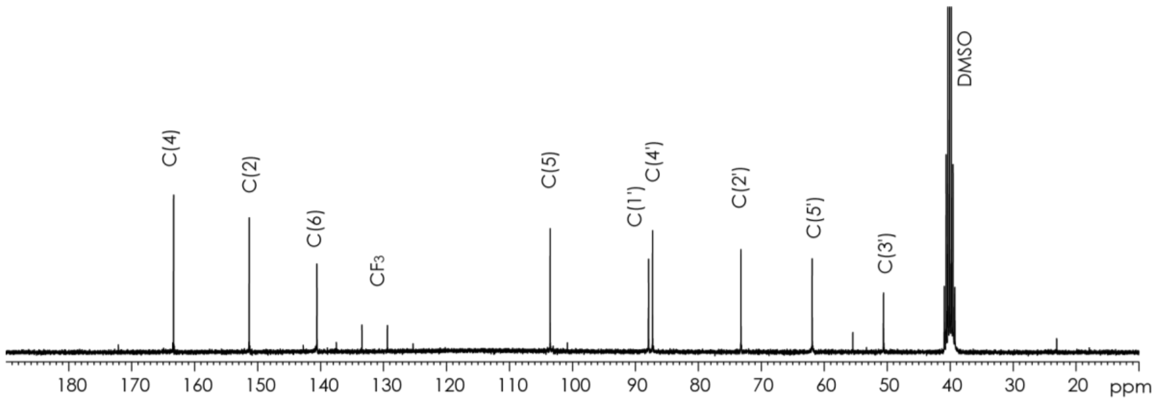

^{19}F-NMR (564 MHz, DMSO) of compound **2**:

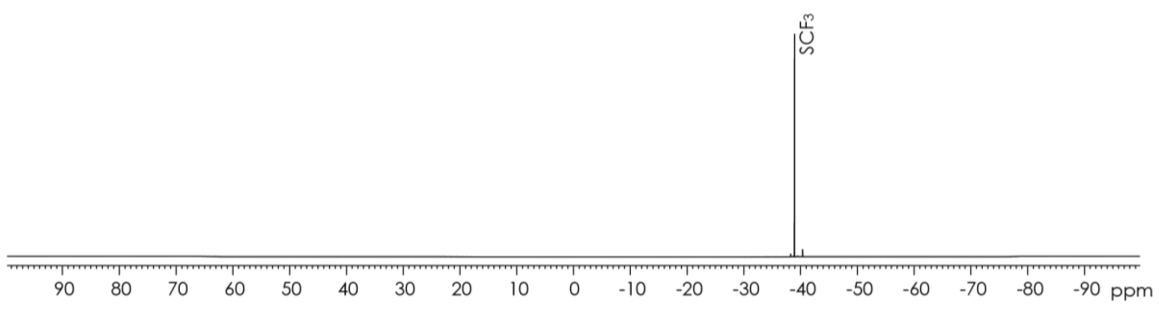

1.2. Synthesis of 5'-*O*-(4,4'-dimethoxytrityl)-2'-deoxy-2'-trifluoromethylthiouridine (3)

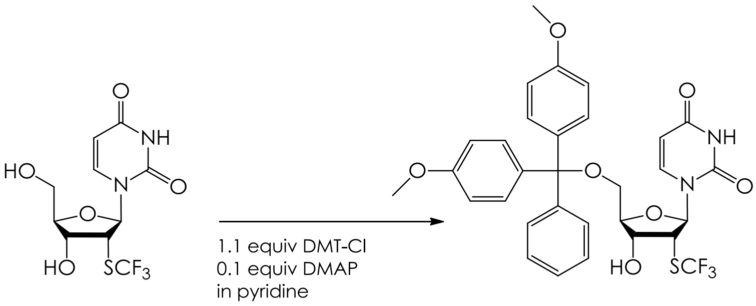

2 $C_{10}H_{11}F_3N_2O_5S$ [328.26]

3 $C_{31}H_{29}F_3N_2O_7S$ [630.63]

5'-*O*-(4,4'-dimethoxytrityl)-2'-deoxy-2'-trifluoromethylthiouridine (**3**). Compound **2** (229 mg, 0.70 mmol) was coevaporated with dry pyridine and dissolved in dry pyridine (5 ml). 4-(Dimethylamino)pyridine (8.5 mg, 0.07 mmol) was added, then the solution was treated with 4,4′-dimethoxytrityl chloride (260 mg, 0.77 mmol) in three portions over 1 hour, followed by stirring overnight at room temperature. The reaction was quenched with a few drops of methanol and the solvents were evaporated. The residue was dissolved in dichloromethane and washed with 5% citric acid, water and saturated sodium bicarbonate solution, dried over $NaSO_4$ and evaporated to dryness. The crude product was purified by column chromatography on silica gel ($CH_2Cl_2/CH_3OH/NEt_3$, 99/0/1 – 97/2/1 v/v/v). Yield: 357 mg of **3** as white foam (81%). TLC (CH_2Cl_2/CH_3OH, 95/5): R_f = 0.29. ^{1}H-NMR (300 MHz, $CDCl_3$): δ 3.52 (s, 2H, 2 x C(5')-H); 3.82 (s, 6H, 2 x OCH_3 DMT); 4.09 (q, *J* = 4.6 Hz, 1H, C(2')-H); 3.28 (s, 1H, C(4')-H); 4.62 (d, *J* = 4.5 Hz, 1H, C(3')-H); 5.36 (d, *J* = 8.1 Hz, 1H, C(5)-H); 6.38 (d, *J* = 9.3 Hz, 1H, C(1')-H); 6.87 (d, *J* = 8.6 Hz, 4H, C(ar)-H DMT); 7.23-7.34 (m, 9H, C(ar)-H DMT); 7.69(d, *J* = 8.1 Hz, 1H, C(6)-H). ^{13}C-NMR (75 MHz, $CDCl_3$): δ 51.86 (C(2')); 55.40 (2 x OCH_3 DMT); 63.89 (C(5')); 73.89 (C(3')); 85.59 (C(4')); 86.54 (C(1')); 87.78 (C(quat.) DMT); 103.43 (C(5)); 113.57, 127.54, 128.22, 128.29 (C(ar)) DMT; 128.50 (CF_3); 130.20, 130.27 (C(ar)) DMT;134.84; 135.03; 139.74 (C(6)); 144.05; 150.86; 158.99; 159.02; 163.06 ppm. ^{19}F-NMR (564 MHz, $CDCl_3$): δ -39.84 (SCF_3) ppm. FT ICR ESI-MS (m/z): $[M+Na]^+$ calcd 653.153 98; found 653.154 75.

^{1}H-NMR (300 MHz, $CDCl_3$) of compound **3**:

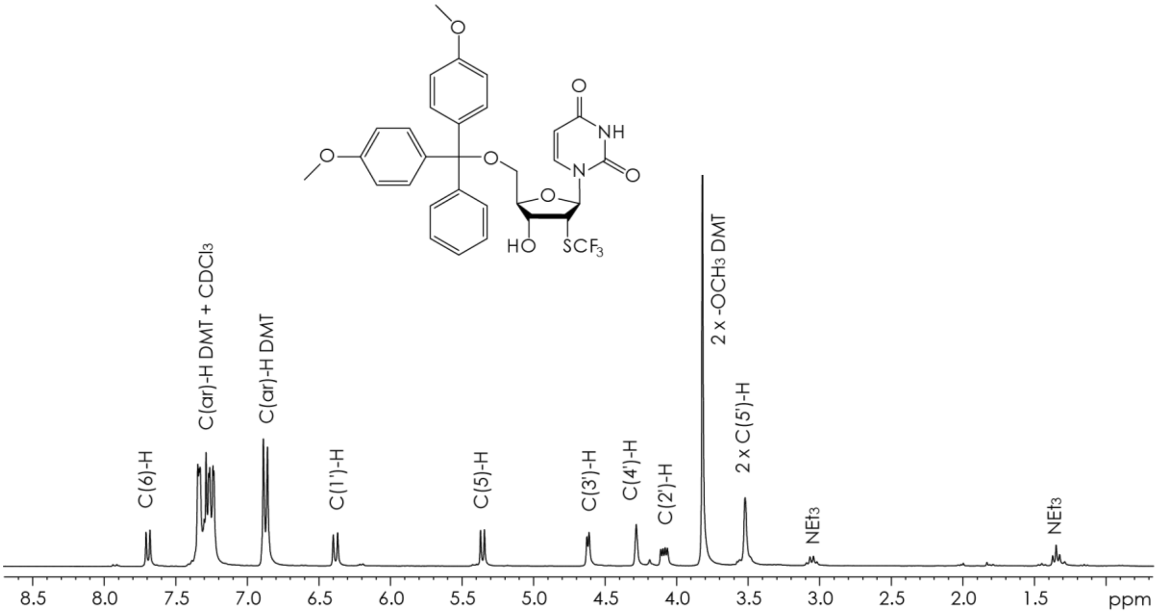

^{13}C-NMR (75 MHz, $CDCl_3$) of compound **3**:

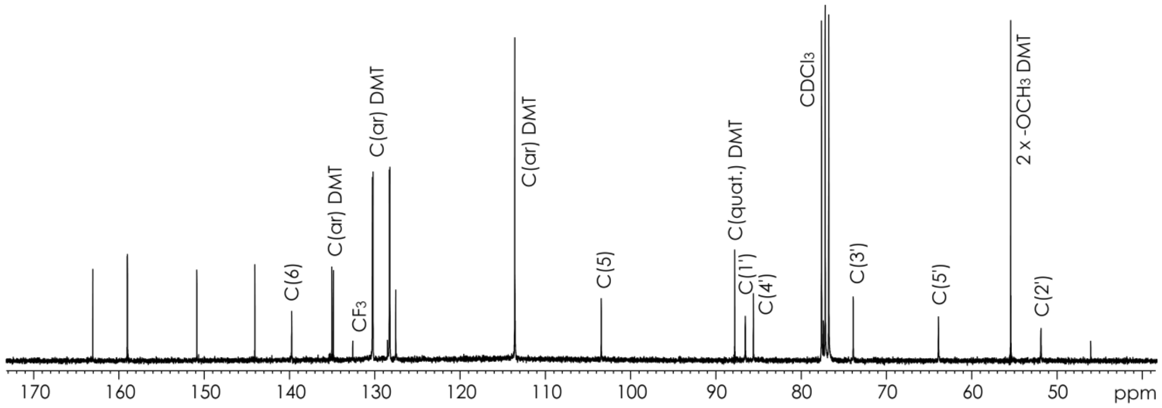

^{19}F-NMR (564 MHz, $CDCl_3$) of compound **3**:

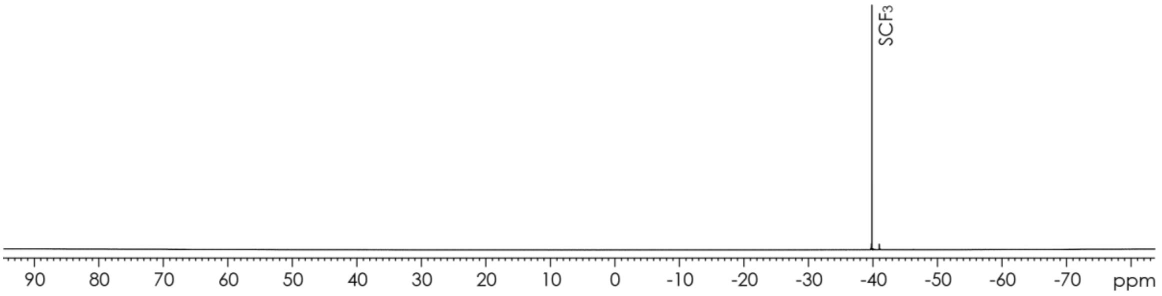

2.3. Synthesis of 5'-*O*-(4,4'-Dimethoxytrityl)-2'-deoxy-2'-trifluoromethylthiouridine 3'-*O*-(2-cyanoethyl *N,N*-diisopropylphosphoramidite) (4)

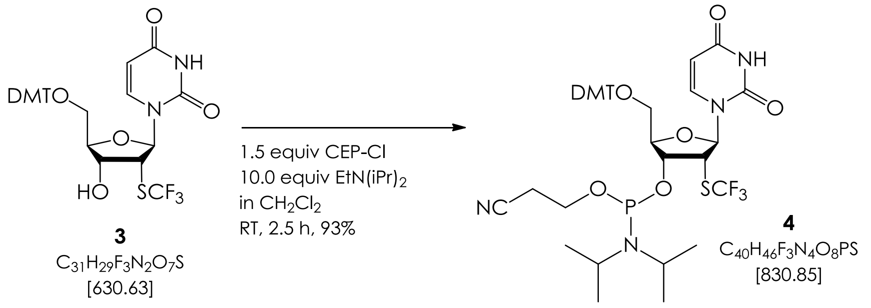

5'-*O*-(4,4'-Dimethoxytrityl)-2'-deoxy-2'-trifluoromethylthiouridine 3'-*O*-(2-cyanoethyl *N,N*-diisopropyl phosphoramidite) (**4**). A solution of compound **3** (220 mg, 0.35 mmol) in dry dichloromethane (1 ml) was treated consecutively with ethyldimethylamine (378 µL, 3.49 mmol) and 2-cyanoethyl diisopropyl phoshoramidochloridite (124 µL, 0.52 mmol). The solution was stirred for 2.5 hours at room temperature under argon atmosphere. After completion of the reaction it was quenched with methanol, diluted with dichloromethane and extracted twice with half saturated sodium bicarbonate solution. The combined organic layers were dried over Na_2SO_4 and evaporated. The crude product was purified by column chromatography on silica gel (ethylacetate/hexanes, 3/7 v/v + 1% NEt_3 – 6/4 v/v + 1% NEt_3). Yield: 269 mg of **4** as white foam (93%). TLC (ethylacetate/hexanes, 5/5): Rf = 0.51. <u>^{1}H-NMR</u> (300 MHz, $CDCl_3$): δ 1.10-1.36 (m, 12H, $((CH_3)_2CH)_2N$); 2.67 (t, *J* = 6.2 Hz, 2H, CH_2CN); 3.49 (s, 2H, 2 x C(5')-H); 3.55-3.77 (m,2H, $((CH_3)_2CH)_2N$); 3.82 (s, 6H, 2 x OCH_3 DMT); 4.88, 4.95 (2d, *J* = 2.2 Hz, 2H, $POCH_2$); 4.06-4.19(m, 1H, C(2')-H); 4.32, 4.41 (s, 1H, C(4')-H); 4.63, 4.79(q, *J* = 4.8 Hz, 1H, C(3')-H); 5.35 (m, 1H, C(5)-H);6.41 (t, *J* = 8.9 Hz, 1H, C(1')-H); 6.88 (d, *J* = 8.6 Hz, 4H, C(ar)-H DMT); 7.24-7.35 (m, 9H, C(ar)-H DMT); 7.69(m, 1H, C(6)-H). <u>^{31}P-NMR</u> (121 MHz, $CDCl_3$): δ 151.35, 152.57. <u>^{19}F-NMR</u> (564 MHz, $CDCl_3$): δ -39.68, -39.79 (SCF_3) ppm. <u>FT ICR ESI-MS</u> (m/z): $[M-H]^-$ calcd 829.264 23; found 829.265 03.

^{1}H-NMR (300 MHz, $CDCl_3$) of compound **4**:

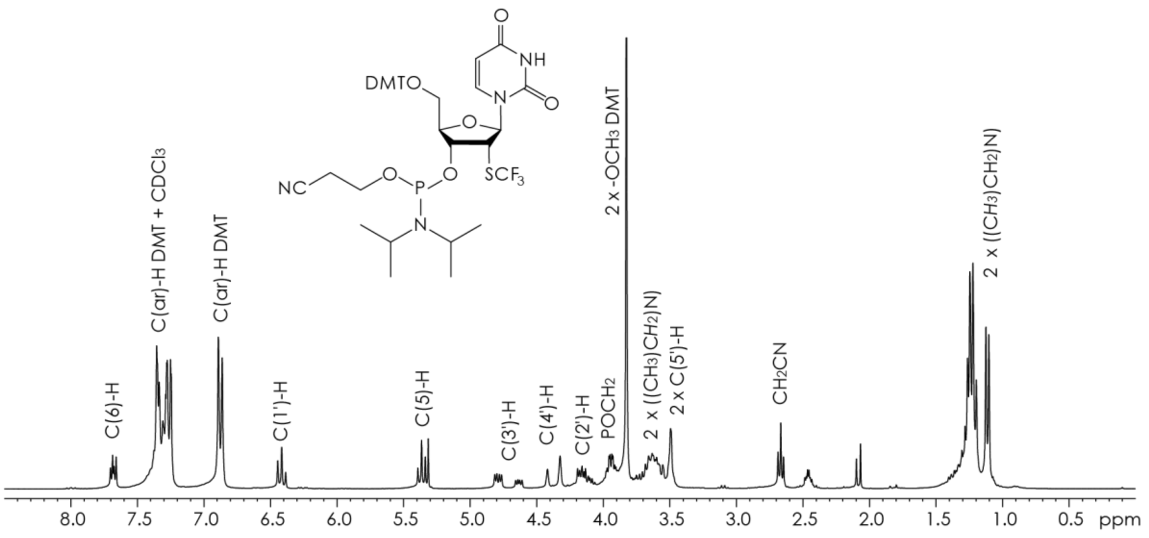

^{13}C-NMR (75 MHz, $CDCl_3$) of compound **4**:

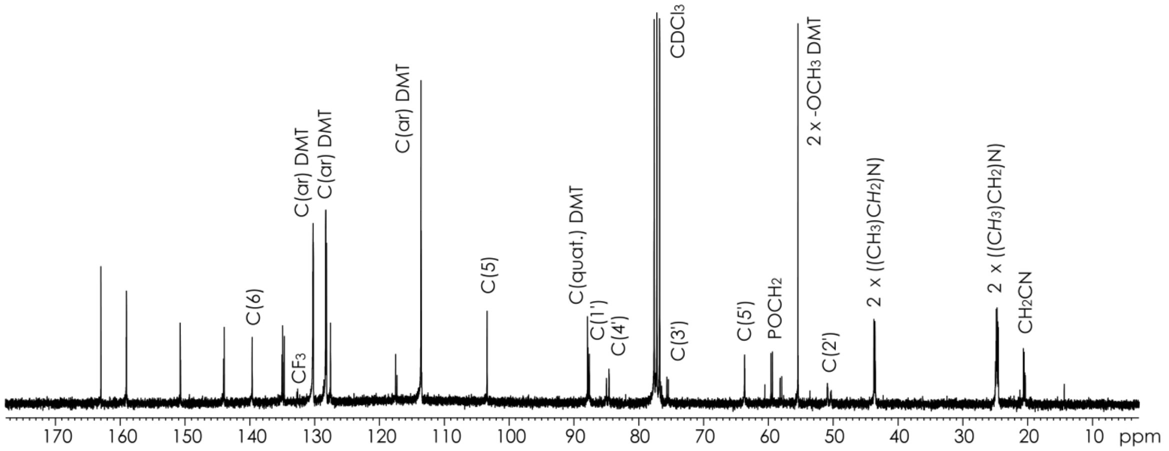

^{19}F-NMR (564 MHz, $CDCl_3$) of compound **4**:

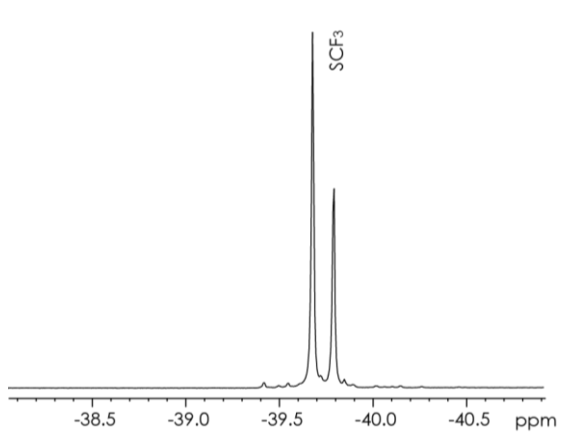

^{31}P-NMR (121 MHz, $CDCl_3$) of compound **4**:

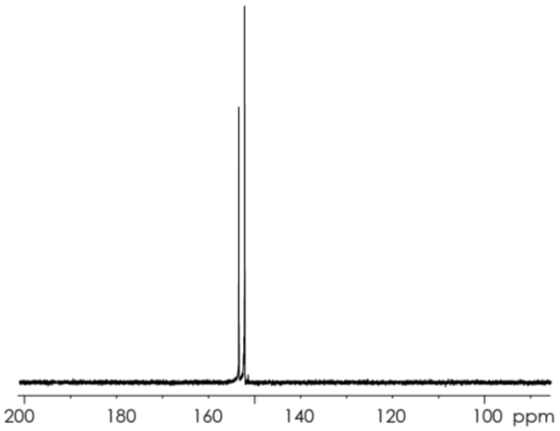

2. RNA synthesis, purification, and MS-, NMR-, and UV-spectroscopic measurements

RNA solid-phase synthesis.

Standard phosphoramidite chemistry was applied for RNA strand elongation. For synthesis, 2'-*O*-TOM standard RNA nucleoside phosphoramidite building blocks were purchased from GlenResearch or ChemGenes; polystyrene supports were purchased from GE Healthcare (Custom Primer SupportTM, 80 µmol/g; PS 200). All oligonucleotides were synthesized on an ABI 392 Nucleic Acid Synthesizer following standard methods: detritylation (80 sec) with dichloroacetic acid/1,2-dichloroethane (4/96); coupling (2.0 min) with phosphoramidites/acetonitrile (0.1 M x 130 µL) and benzylthiotetrazole/acetonitrile (0.3 M x 360 µL); capping (3 x 0.4 min, Cap A/Cap B =1/1) with Cap A: 4-(dimethylamino)pyridine in acetonitrile (0.5 M) and Cap B: Ac_2O/*sym*-collidine/acetonitrile (2/3/5); oxidation (1.0 min) with I_2 (20 mM) in THF/pyridine/H_2O (35/10/5). The solutions of amidites, tetrazole and acetonitrile were dried over activated molecular sieves (4 Å) overnight.

Deprotection of 2'-SCF_3 modified RNA.

The solid support was dried and treated with $MeNH_2$ in EtOH (33%, 0.5 mL) and $MeNH_2$ in water (40%, 0.5 mL) for 6-8 h at room temperature. The supernatant was removed and the solid support was washed 3 x with THF/water (1/1, v/v). The supernatant and the washings were combined and the whole mixture was evaporated to dryness. To remove the 2'-silyl protecting groups, the resulting residue was treated with tetrabutylammonium fluoride trihydrate (TBAF·$3H_2O$) in THF (1 M, 1 mL) at 37°C overnight. The reaction was quenched by the addition of triethylammonium acetate (TEAA) (1 M, pH 7.4, 1 mL). The volume of the solution was reduced and the solution was desalted on a size exclusion column (GE Healthcare, HiPrep™ 26/10 Desalting; 2.6 x 10 cm; Sephadex G25) eluating with H_2O, the collected fraction was evaporated to dryness and dissolved in 1 ml H_2O. Analysis of the crude RNA after deprotection was performed by anion-exchange chromatography on a Dionex DNAPac® PA-100 column (4 mm x 250 mm) at 80°C. Flow rate: 1 mL/min, eluant A: 25mM Tris·HCl (pH 8.0), 6 M urea; eluant B: 25 mM Tris·HCl (pH 8.0), 0.5 M $NaClO_4$, 6 M urea; gradient: 0-60 % B in A within 45 min or 0-40 % B in 30 min for short sequences up to 15 nucleotides, UV-detection at 260 nm.

Purification of 2'-SCF_3 uridine modified RNA.

Crude RNA products were purified on a semipreparative Dionex DNAPac® PA-100 column (9 mm x 250 mm) at 80°C with flow rate 2 ml/min. Fractions containing RNA were loaded on a C18 SepPak Plus® cartridge (Waters/Millipore), washed with 0.1-0.15 M $(Et_3NH)^+HCO_3^-$, H_2O and eluted with H_2O/CH_3CN (1/1). RNA containing fractions were lyophilized. Analysis of the quality of purified RNA was performed by analytical anion-exchange chromatography (for conditions see paragraph above). The molecular weights of all synthesized RNAs were confirmed by LC-ESI mass spectrometry. Yields were determined by UV photometrical analysis of oligonucleotide solutions.

Mass spectrometry of 2'-SCF_3 uridine modified RNA.

All experiments were performed on a Finnigan LCQ Advantage MAX ion trap instrumentation connected to an Amersham Ettan micro LC system. RNA sequences were analyzed in the negative-ion mode with a potential of -4 kV applied to the spray needle. LC: Sample (200 pmol RNA dissolved in 30 µL of 20 mM EDTA solution; average injection volume: 30 µL); column (Waters XTerra®MS, C18 2.5 µm; 1.0 x

50 mm) at 21°C; flow rate: 30 µL/min; eluant A: 8.6 mM TEA, 100 mM 1,1,1,3,3,3-hexafluoroisopropanol in H_2O (pH 8.0); eluant B: methanol; gradient: 0-100 % B in A within 30 min; UV-detection at 254 nm.

^{19}F-and ^{1}H-NMR spectroscopy of 2'-SCF_3 uridine RNA.

The RNA sample was lyophilized and dissolved in 25 mM Na_2HAsO_4 buffer pH 7.0 (450 µl) and D_2O (50 µl) was added to a total volume of 500 µL. Final concentrations were as indicated in the corresponding figure captions. All samples were heated to 90 °C for 1 min, then rapidly cooled in an ice bath and equilibrated to room temperature for 15 min before measurements.

The ^{1}H-1D-NMR spectra were acquired using a double-pulsed field gradient spin-echo pulse sequence. ^{19}F NMR spectra were recorded at a frequency of 564.6 MHz on a Bruker Avance II+ 600 MHz spectrometer equipped with a 5 mm QNP probe with Z-gradient (^{2}H/^{1}H/^{19}F). Typical experimental parameters were chosen as follows: spectral width 20 ppm, ^{19}F 90°-pulse 13 µs, acquisition time 1.2 s, relaxation delay 1.5 - 2.0 s. Prior to Fourier transformation all time domain data was processed with an exponential window function using a line broadening factor of 2-4 Hz. ^{19}F resonances are reported relative to external CCl_3F.

<u>Prepraration of *E.coli* cell lysate</u>:

The cell lysate was prepared by growing *E. coli* cells (strain BL21(DE3)) overnight in 50 ml LB medium (37 °C, 180 rpm). The final OD^{600} was 1.87. The cells were harvested by centrifugation at 4 °C. Excess salts were removed by resuspending the cell pellet in 20 ml sterile water followed by centrifugation at 4 °C (2 x). Finally, the cells were resuspended in sterile water (25 mL) and then lysed by ultra sonic treatment. Cellular debris was removed by centrifugation and the supernatant was transferred and filled up to a final volume of 50 ml using sterile water. The lysate was stored at -80°C up to one week. For NMR sample preparation, the RNA was dissolved in undiluted lysate and 10% of D_2O was added.

Thermal denaturation studies

Absorbance versus temperature profiles were recorded at 250 nm and 260 nm on a Varian Cary 100 spectrophotometer equipped with a multiple cell holder and a Peltier temperature-control device. Data were collected after a complete cooling and heating cycle at a rate of 0.7°C/minute. Sample preparation: Oligonucleotides were lyophilized to dryness, dissolved in melting buffer (10 mM Na_2HPO_4, 150 mM NaCl, pH 7.0) and subsequently degassed. A layer of silicon oil was placed on the surface of the solution. Values of ΔH^0 and ΔS^0 for monomolecular melting transitions were derived from a two-state van't Hoff analysis by fitting the shape of the individual α versus temperature curve. For bimolecular melting transitions the values of ΔH^0 and ΔS^0 were derived from 1/T versus ln(c) plotting as described in literature.[2]

Supporting Table 1. Selection of 2'-SCF_3 modified RNA.

			**Molecular weight** [amu]	
**sequence (5'→3')**	**nt**	**Yield** [nmol]	**calc**	**found**
UGU $U_{2'SCF_3}$GC	6	1419.4	1936.19	1935.84
CGC C$U_{2'SCF_3}$U CC	8	907.3	2505.53	2505.69
GG$U_{2'SCF_3}$ CGA CC	8	507.9	2608.84	2608.63
GAA GGG CAA CC$U_{2'SCF_3}$ UCG	15	513.3	4898.03	4898.14
CGG AAG GUC CGC C$U_{2'SCF_3}$U CC	17	316.5	5461.32	5461.44
GGG CGA UUU U$U_{2'SCF_3}$A UCG CU	17	541.5	5465.28	5465.49
GAC CGG AAG GUC CGC C$U_{2'SCF_3}$U CC	20	449.2	6440.92	6441.20
UAU CCA UUG CAC $U_{2'SCF_3}$CC GGA UG	20	373.9	6387.86	6388.16
UAU CCA UUG CAC C$U_{2'SCF_3}$C GGA UG	20	207.3	6388.85	6389.41
UAU CCA $U_{2'SCF_3}$UG CAC UCC GGA UG	20	352.2	6387.86	6388.34
GCG UGG UUU AGG UAA $U_{2'SCF_3}$GC GCU ACC UAA	27	279.5	8734.28	8735.02
GCG $U_{2'SCF_3}$GG UUU AGG UAA UGC GCU ACC UAA	27	170.4	8734.28	8735.08
UGC $U_{2'SCF_3}$CC UAG UAC GAG AGG ACC GGA GUG	27	258.0	8811.37	8811.82
UGC UCC UAG $U_{2'SCF_3}$AC GAG AGG ACC GGA GUG	27	331.0	8811.37	8811.73
GGC GUU UUC GCC UUC GGG CGA UUU U$U_{2'SCF_3}$A UCG CU	32	270.2	10209.04	10209.21

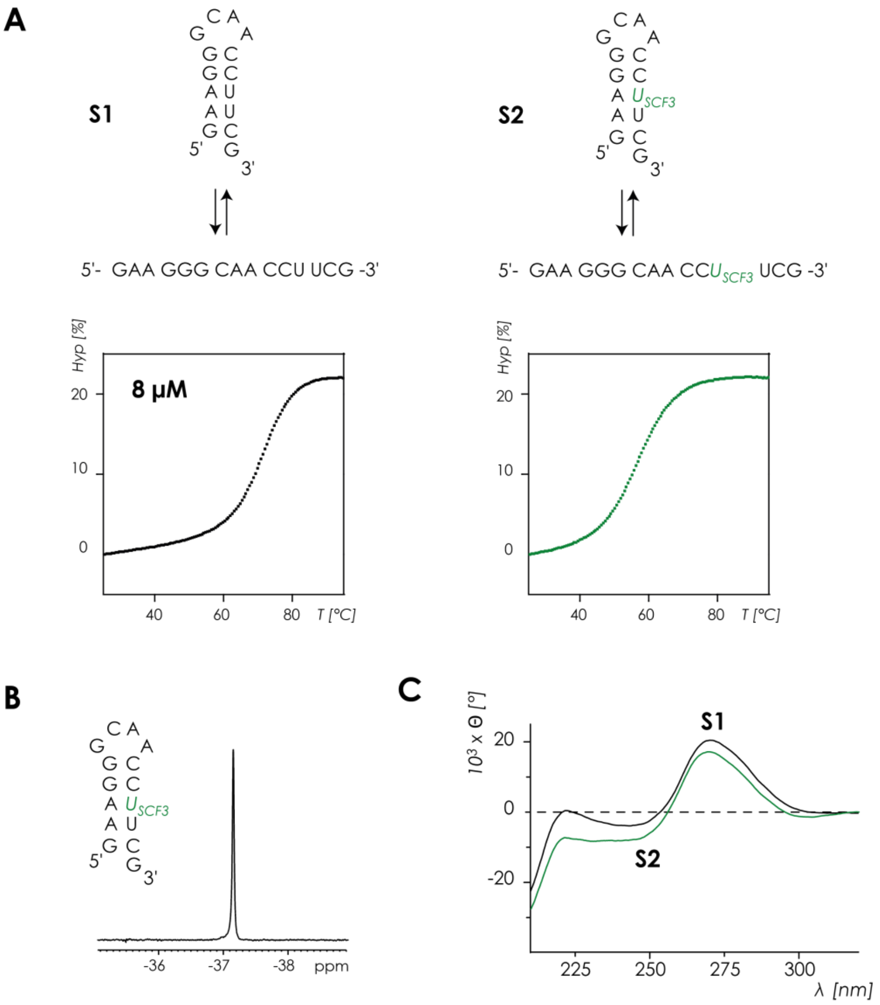

Supporting Figure 1. A) Comparison of thermal denaturation of unmodified 5'-GAA GGG CAA CCU UCG-3' (**S1**) and 2'-SCF_3 uridine modified 5'-GAA GGG CAA C**C**$_{2'SCF3}$U UCG-3' (**S2**) hairpin forming RNA sequences (c_{RNA} = 8 µM). Shown: Hyperchromicities (250 nm) as function of the temperature; buffer conditions: 10 mM $Na_2HPO_4 \cdot 7H_2O$, 150 mM NaCl, pH 7.0. B) ^{19}F-NMR of the modified sequence **S2** shows a single signal for the 2'-SCF_3-uridine modified hairpin-forming sequence. C) Overlay of CD-spectra of **S1** and **S2**, conditions: Jasco 403 CD-spectrophotometer, 210-320 nm, step resolution 0.2 nm, speed 100, accumulation 3, bandwith 2.0, sensitivity 10 mdeg; T = 18°C, buffer: 10 mM $Na_2HPO_4 \cdot 7H_2O$, 150 mM NaCl Puffer, pH 7.0; c_{RNA}=3 µM.

Supporting Table 2. Thermal and thermodynamic analysis of unmodified and 2'-SCF_3 modified RNAs.[a]

RNA	c [µM]	T_m [°C]	RNA	c [µM]	T_m [°C]	ΔT_m [°C]
**S1**	2	**72.2**	**S2**	2	**57.0**	**15.2**
	4	**72.2**		4	**57.7**	**14.5**
	8	**72.2**		8	**56.9**	**15.3**
	16	**70.6**		16	**57.4**	**13.2**
	32	**72.4**		32	**57.7**	**14.7**

RNA	T_m [°C]	ΔT_m [°C]	$\Delta H°$ [kcal·mol^{-1}]	$\Delta S°$ [cal·mol^{-1} K^{-1}]	$\Delta G°_{298K}$ [kcal·mol^{-1}]
**S1**	71.9 ± 0.7	14.6	-51.64	-150.24	**-6.8**
**S2**	57.3 ± 0.4		-50.01	-151.25	**-4.9**

[a] RNA sequences: 5'-GAA GGG CAA CCU UCG-3' **S1** and 5'-GAA GGG CAA CC(**2'-SCF_3-U**) UCG-3' **S2**. The thermodynamic parameters for monomolecular melting transitions were calculated from the melting profiles. The values of $\Delta H°$, $\Delta S°$ and $\Delta G°$ were derived from a two-state van't Hoff analysis by fitting the shape of the individual α versus temperature curve as described in the literature.[2]

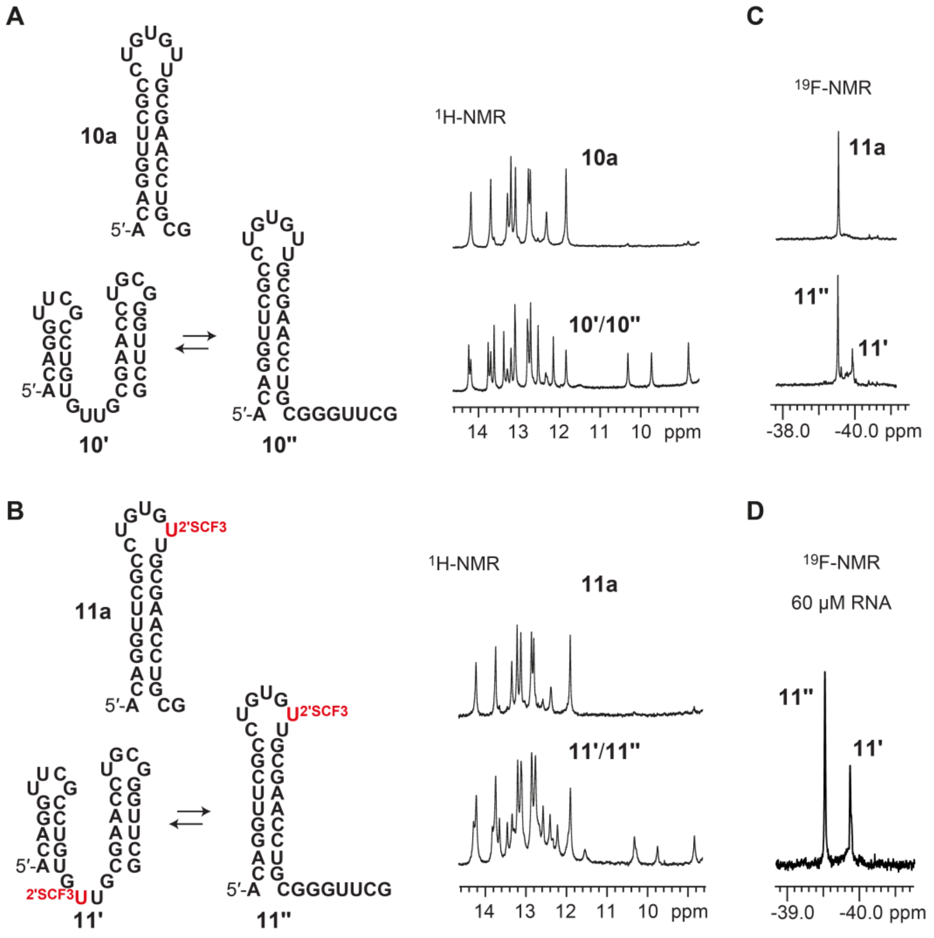

Supporting Figure 2. Structure probing of bistable RNA. A) Unmodified RNA;[3] secondary structure model of full-length (**10'**, **10"**) and reference (**10a**) RNA (left); ^{1}H imino proton NMR spectra (right). B) Same as (A), but for 2'-SCF_3 labeled analogues. C) Assignment of folds **11'** and **11"** of RNA **11** by ^{19}F-NMR spectroscopy. D) Same as C, but c_{RNA} = 60 µM. Conditions for A-C: c_{RNA} = 0.3 mM, 25 mM Na_2HAsO_4, pH 7.0, H_2O/D_2O 9/1, 298 K.

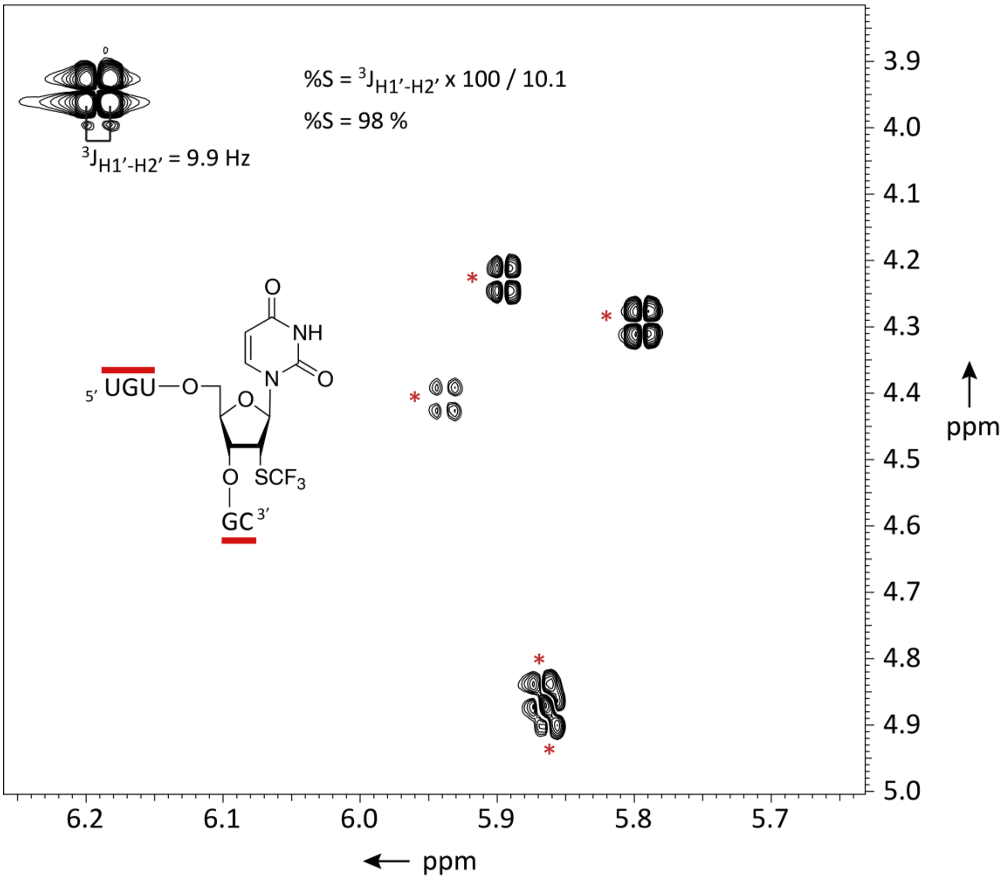

Supporting Figure 3. DQF-COSY of a 6 nucleotide RNA sequence containing a 2'-SCF_3 modification. The 3-bond scalar coupling constants of H1' and H2' ($^3J_{H1'-H2'}$) were extracted from the corresponding crosspeaks. For the 2'-SCF_3-uridine moiety, a coupling constant of 9.9 Hz was determined. Assuming a pure C2'/C3'-endo equilibrium, this value is correlated to a C2'-endo population of 98%.[4],[5] For the other single-stranded standard RNA residues, coupling constants of 8.5 to 9.0 Hz were measured corresponding to C2'-endo populations between 84 to 89%.

4. References:

[1] a) Imazawa, M.; Ueda, T.; Ukita, T. Synthesis of 2′-deoxy-2′-mercaptouridine. *Tetrahedron Lett.* 1970, *11*, 4807-4810; b) Divakar, K. J.; Mottoh, A.; Reese, C. B., et al. Approaches to the synthesis of 2'-thio analogues of pyrimidine ribosides. *J. Chem. Soc., Perkin Trans. 1* 1990, 969-974; c) Le Hir de Fallois, L.; Decout, J.-L.; Fontecave, M. Synthesis of 2'-thio-uridine and -cytidine derivatives as potential inhibitors of ribonucleoside diphosphate reductase: thionitrites, disulfides and 2'-thiouridine 5'-diphosphate. *J. Chem. Soc., Perkin Trans. 1* 1997, 2587-2596.

[2] a) Marky, L. A.; Breslauer, K. J. Calculating thermodynamic data for transitions of any molecularity from equilibrium melting curves. *Biopolymers* 1987, 26, 1601-1620; b) Xia, T., Mathews, D. H., Turner, D. H. Thermodynamics of RNA secondary structure formation. *In Comprehensive Natural Product Chemistry*, Vol. 8 (Ed. Söll, D.; Nishimura, S., Moore, P.), Elsevier, Oxford, 1999, 21-47.

[3] Micura, R.; Höbartner, C. On secondary structure rearrangements and equilibria of small RNAs. *ChemBioChem* 2003, *4*, 984-990.

[4] Haziri A. I., Leumann C. J. Synthesis and pairing properties of oligodeoxynucleotides containing bicyclo-RNA and bicyclo-ANA modifications. *JOC* 2012, *77*, 5861-5869.

[5] Altona C., Sundaralingam M. J. Conformational analysis of the sugar ring in nucleosides and nucleotides. Improved method for the interpretation of proton magnetic resonance coupling constants. *J. Am. Chem Soc.* 1973, *95*, 2333-2344.
